# Is immune checkpoint modulation a potential therapeutic option in triple negative breast cancer?

**DOI:** 10.1186/s13058-014-0457-z

**Published:** 2014-11-07

**Authors:** David J Klinke

**Affiliations:** 1 Department of Chemical Engineering and Mary Babb Randolph Cancer Center, West Virginia University, Morgantown, 25606 WV USA; 2 Department of Microbiology, Immunology, and Cell Biology, West Virginia University, Morgantown, 25606 WV USA

## Abstract

No abstract.

**Electronic supplementary material:**

The online version of this article (doi:10.1186/s13058-014-0457-z) contains supplementary material, which is available to authorized users.

The emergence of molecular targeted therapies has revolutionized the clinical treatment of breast cancer. To guide treatment, patient samples are screened for expression of hormone receptors for estrogen and progesterone and the epidermal growth factor receptor HER2. Patients with tumors that do not express any of these three receptors (that is, triple-negative breast cancer) exhibit a worse outcome [1]. Sequencing of cancer genomes suggests that over-expressing an oncogene or eliminating a tumor suppressor gene is also associated with passenger mutations. The presence of these passenger mutations may provide a collective signature that distinguishes malignant from normal cells. Conceptually, the adaptive immune system recognizes cells that present a different antigenic signature and provides a mechanism to control for malignant transformation. One approach to enhance anti-tumor immunity is to increase the number of T cells, either systemically, through inhibiting the action of CTLA-4, or locally, through inhibiting the programmed cell death 1 pathway. Therapeutic inhibition of these pathways is called immune checkpoint modulation [2]. The clinical benefit received by a subset of patients with metastatic melanoma demonstrates proof-of-principle for this therapeutic approach [3].

In a retrospective study of invasive breast cancer, we found that increased expression of genes associated with type 1 immunity was a predictor of increased survival independent of molecular pathology [4]. This gene signature includes type 1 T-cell polarization and enhanced cytotoxic T-cell and natural killer cell recruitment. While this finding is consistent with a number of other studies (for example, [5]), we also found that 70% of patients with invasive triple-negative breast cancer clustered with the cohort characterized by an increased type 1 immune signature. In examining the gene expression signature, we found that the expression of several type 1 immunity genes aligned along the direction of principal coordinate 1 (Figure 1A). In addition, expression of members of the programmed cell death 1 pathway (*PDCD1* and *CD274*) that can be therapeutically targeted also aligned in the same direction (Figure 1B). Other genes typically associated with local immunosuppression, including *TGFB1*, *MICB/MICA*, *HMGB1*, *HIF1A*, *FOXP3*, and *IL10*, were not significantly different than random noise. Collectively, these findings suggest two points: first, patients with invasive triple-negative breast cancer have an increased propensity for on-going anti-tumor immunity; and second, therapeutic relief of the programmed cell death 1 pathway may improve overall survival in patients with triple-negative breast cancer.

**Figure 1:**
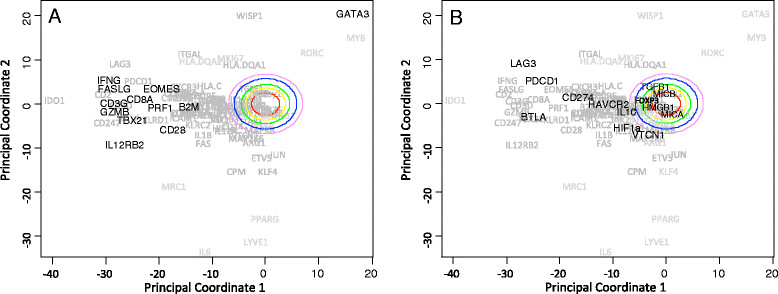
**Expression of PD-1 (PDCD1) and PD-L1 (CD274) mRNA within tumor samples correlates with a type 1 immune gene signature.** Principal coordinate analysis was applied to expression data for a subset of immune-related genes obtained from the invasive breast cancer arm of the Cancer Genome Atlas (Figure S3 in [4]). Biplot projections of the genes along the first two principal coordinate directions, where principal coordinate 1 corresponds to a type 1 immune signature and principal coordinate 2 corresponds to oncogenic transformation in invasive breast cancer. Type 1 immune related genes are shown in black in **(A)** while immunosuppressive genes are highlighted in **(B)**. The first two principal coordinates capture 33% of the overall variance in the data. As principal coordinates are independent, the projection of a gene along the corresponding axes indicates the degree to which the expression of two genes are related and the distance from the origin indicates the strength of the covariation within the data set. The remaining principal coordinates capture progressively less variance in the data and provide little additional information. The colored ovals radiating out from the origin indicate principal coordinate values that cannot be distinguished from random noise, that is, a null hypothesis, with increasing levels of statistical stringency. These colored ovals were obtained by bootstrap resampling.

## Authors' contributions

DJK conceived the study, performed the bioinformatic analysis, analyzed the experimental data, and wrote the manuscript.

## Authors’ original submitted files for images

Below are the links to the authors’ original submitted files for images. Authors’ original file for figure 1
