# Supplementary figures and images for: Is immune checkpoint modulation a potential therapeutic option in triple negative breast cancer?
Source: Breast Cancer Res. 2014 Nov 7;16:457. doi: 10.1186/s13058-014-0457-z (PMC4303193; doi:10.1186/s13058-014-0457-z)

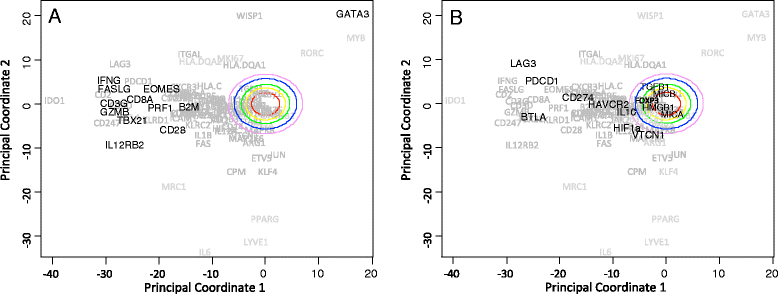

Supplement: Supplementary file 1 — Authors’ original file for figure 1 [file 13058_2014_457_MOESM1_ESM.gif]
